# Recent Progress in Amaryllidaceae Biotechnology

**DOI:** 10.3390/molecules25204670

**Published:** 2020-10-13

**Authors:** Vasil Georgiev, Ivan Ivanov, Atanas Pavlov

**Affiliations:** 1 Laboratory of Cell Biosystems, The Stephan Angeloff Institute of Microbiology, Bulgarian Academy of Sciences, Plovdiv 4000, Bulgaria; vasgeorgiev@gmail.com; 2 Department of Organic Chemistry and Inorganic Chemistry, University of Food Technologies, Plovdiv 4002, Bulgaria; ivanov_ivan.1979@yahoo.com; 3 Department of Analytical Chemistry and Physical Chemistry, University of Food Technologies, Plovdiv 4002, Bulgaria

**Keywords:** alkaloids, Amaryllidaceae, bioreactors, galanthamine, plant in vitro systems

## Abstract

Plants belonging to the monocotyledonous Amaryllidaceae family include about 1100 species divided among 75 genera. They are well known as medicinal and ornamental plants, producing pharmaceutically important alkaloids, the most intensively investigated of which are galanthamine and lycorine. Amaryllidaceae alkaloids possess various biological activities, the most important one being their anti-acetylcholinesterase activity, used for the treatment of Alzheimer’s disease. Due to increased demand for Amaryllidaceae alkaloids (mainly galanthamine) and the limited availability of plant sources, in vitro culture technology has attracted the attention of researchers as a prospective alternative for their sustainable production. Plant in vitro systems have been extensively used for continuous, sustainable, and economically viable production of bioactive plant secondary metabolites. Over the past two decades, a significant success has been demonstrated in the development of in vitro systems synthesizing Amaryllidaceae alkaloids. The present review discusses the state of the art of in vitro Amaryllidaceae alkaloids production, summarizing recently documented plant in vitro systems producing them, as well as the authors’ point of view on the development of biotechnological production processes with a focus on the future prospects of in vitro culture technology for the commercial production of these valuable alkaloids.

## 1. Introduction

Plants belonging to the monocotyledonous Amaryllidaceae family include about 1100 species divided among 75 genera (*Amaryllis*, *Galanthus*, *Leucojum*, *Narcissus*, *Haemanthus*, *Nerine*, *Hippeastrum*, *Sternbergia*, *Clivia*, *Rhodophiala*, *Pancratium*, *Hymenocallis*, *Crinum*, *Lycoris*, etc.), which are well known, mainly as medicinal and ornamental plants [1,2]. The Amaryllidaceae family is one of the most important alkaloid-producing plant families [3]. A number of alkaloids grouped in different structural types, such as lycorine, galantamine, haemantamine, homolicorine, tazetine, monthanitne, narciclasine, have been isolated and identified by GC-MS, NMR and HPLC. The most intensively investigated alkaloids are galanthamine and lycorine [4]. Various valuable pharmacological and biological effects of Amaryllidaceae alkaloids have been reported, such as antiviral, antiparasitic, antimalarial, anticancer, antibacterial and antioxidant activities [5,6,7,8,9,10,11,12,13,14,15]. The continuously increasing interest in Amaryllidaceae alkaloids in the last years is based on important pharmaceutical properties. Galanthamine possesses anti-acetylcholinesterase activity, used for the treatment of Alzheimer’s disease, and is approved for use in the European Union and the United States [16]; lycorine possesses cytotoxicity and antitumor properties [17]; montanine possesses anxiolytic, antidepressant and anticonvulsive activities as well as immunomodulatory properties [18]; pancratistatin and narciclasine possess antitumor activity [19,20].

Due to the increased demand for Amaryllidaceae alkaloids (mainly galanthamine) and the limited availability of plant sources, in vitro culture technology has attracted the attention of researchers as a prospective alternative for their sustainable production [21]. Plant in vitro systems have been extensively used for continuous, sustainable, and economically viable production of bioactive plant secondary metabolites [22]. A significant success has been demonstrated in the development of in vitro systems for production of Amaryllidaceae alkaloids [23,24,25,26,27,28]. Optimization of the production process is also well documented, starting with nutrient media optimization [29], development of appropriate bioreactor design [24,30,31], and optimization of the cultivation conditions [27,32,33]. In 2018, Laurain-Mattar and Ptak published a book chapter [28] which is very informative and helpful to scientists working in this area.

This review examines the state of the art of in vitro Amaryllidaceae alkaloid production, summarizing recently documented plant in vitro systems producing them as well as the authors’ point of view on the development of biotechnological production processes with a focus on the future prospects of the in vitro culture technology for the commercial production of these valuable alkaloids.

## 2. Plant In Vitro Systems Producing Amaryllidaceae Alkaloids

Biotechnologies based on plant in vitro systems have been extensively used for the continuous, sustainable, and economically viable production of plant secondary metabolites. Research on plant cell, tissue and organ cultures as potential sources of Amaryllidaceae alkaloids started in 1963 with the pioneering investigations of Fales and co-workers [34], and nowadays the potential for alkaloid production of Amaryllidaceae plant in vitro systems, with different degrees of cell differentiation, is well documented in literature [21,27,34,35,36,37]. The most recent review on the topic is the 2018 book chapter by Laurain-Mattar and Ptak [28]. Recent reports on plant in vitro systems producing Amaryllidaceae alkaloids are summarized in Table 1.

## 3. Biotechnological Production of Amaryllidaceae Alkaloids

The development of biotechnologies for the production of biologically active substances based on plant cells and tissues cultured under in vitro conditions is a complex and multi-stage process. Algorithms for optimizing and controlling the process of biosynthesis of the target metabolite in the in vitro systems under study should be based on the bioengineering, physiological and phytochemical peculiarities of the particular in vitro culture, as well as on the subsequent analysis of the relationships in the “in vitro system–product” biological system [49,50]. A preliminary requirement for industrially significant yields of target biologically active substances from plant in vitro systems is the development of an efficient cultivation system with appropriately designed bioreactors, as well as unconventional strategies for optimization of the biological systems [51,52].

**Figure 1:**
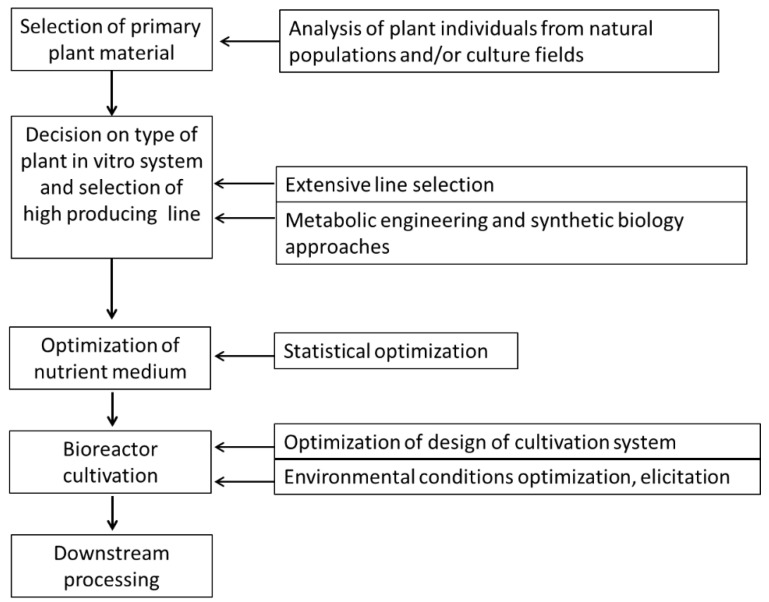
Integrated approach to biotechnological production of Amaryllidaceae alkaloids.

Based on the summarized results of extensive research in this field over a period of more than 20 years, the authors propose the following integrated approach to process development for production of Amaryllidaceae alkaloids by plant in vitro systems (Figure 1).

### 3.1. Selection of Primary Plant Material

It is postulated that the genetic potential of an intact plant from which explants are extracted to produce in vitro systems directly affects the biosynthetic potential of in vitro systems. This fact is often underestimated by plant biotechnologists, although it is the basis for the successful development of technologies for bioactive secondary metabolite production. Diop and co-workers [53] have developed a protocol for obtaining hairy roots from *Leucojum aestivum* L. Obtaining hairy roots from monocotyledonous plant was a great success. Unfortunately, further analyses showed that hairy roots did not biosynthesize galanthamine. However, are the reported results significant for the biosynthetic potential of *L. aestivum* hairy roots? In this case the biosynthetic potential of the obtained hairy roots cannot be estimated, as authors reported that bulbs of the primary plants used did not accumulate galanthamine. It is clear that the selection of the plant individuals further used as primary material for development of a plant in vitro system should be based on systematic screening procedures. Extensive botanical and phytochemical evaluation of the wild populations and/or clone origin of the field crop is required to select the most suitable plant individuals for further in vitro work.

Good examples in this regard are the investigations presented by three groups of Bulgarian scientists. Gussev et al. [54] assessed 22 wild populations of *L. aestivum* L. in Bulgaria in terms of economic potential for galanthamine production, providing valuable information for further selection procedures of plant individuals for in vitro work. Bogdanova and co-workers [55] investigated the influence of the clone origin of field cultivated *L. aestivum* L. plants on the biosynthetic potential of the corresponding in vitro systems and clearly demonstrated the relationship between alkaloid profiles and galanthamine quantity in the intact plants and in vitro systems. Georgieva et al. [45] investigated the alkaloid variability in *L. aestivum* L. from wild populations and concluded that the alkaloid profiles and the quantity of major alkaloids obtained from intact plants and in vitro system were similar. In our opinion, there is a need of more thorough investigations on the intrapolulation variations of alkaloids in wild habitats in order to develop and validate procedures for selecting the most productive plant individuals for further introduction in vitro. The intrapopulation variability observed in alkaloid content could be due to genotypic variability of the population and to meta-genomic changes as well.

### 3.2. Decision on the Type of Plant In Vitro System

Data presented in Table 1 clearly show that undifferentiated in vitro systems (callus) derived from Amaryllidacea species exhibit low biosynthetic potential with galanthamine accumulated in *Narcissus* ssp. from 0 to 7 µg/g [22,38,39], and higher concentrations from 10 to 13 µg/g DW were reported for *L. aestivum* [36,44]. Similar results were obtained for the biosynthesis of lycorine from undifferentiated callus cultures [38,39,41,44]. These low yields of the target alkaloids indicate that undifferentiated tissue cultures are inappropriate for developing successful and highly productive biotechnology for their production. As a counterpoint, differentiated plant in vitro systems of *Narcissus pseudonarcissus*, *Narcissus tazzetta* and *L. aestivum* (Table 1) accumulated more than 30–40-fold higher concentrations of Amarillydacea alkaloids [22,36,38,39,44,56]. This fact leaves open many questions regarding the scale-up of cultivation processes, because the difficulties associated with bioreactor cultivation of differentiated plant in vitro systems are well known [57].

### 3.3. Metabolic Engineering and Synthetic Biology Approaches to Increase the Production of Amaryllidaceae Alkaloids

**Figure 2:**
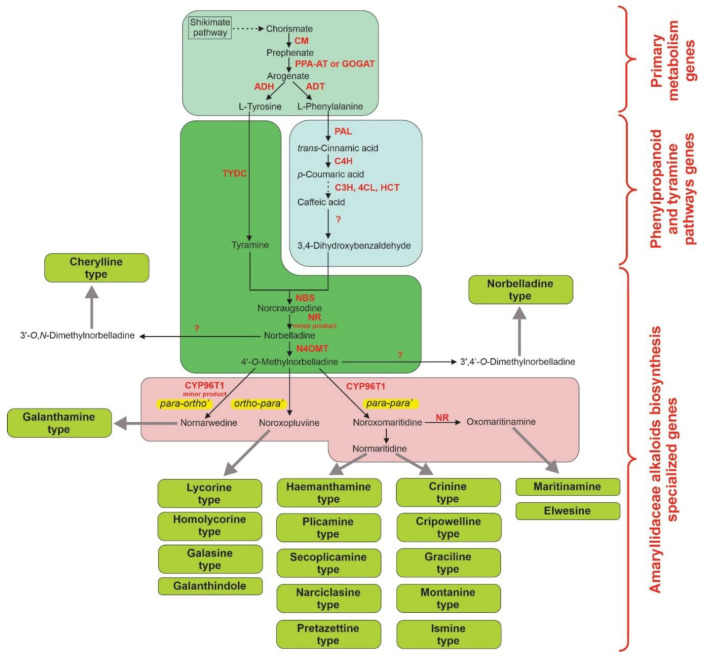
Schematic presentation of the proposed biosynthetic pathway of Amaryllidaceae alkaloid biosynthesis in plants. The five stages of the pathway are presented in different colors. The dashed arrows represent multiple reactions. The bold grey arrows indicate specialized pathways leading to the biosynthesis of different types of Amaryllidaceae alkaloids. The known enzymes are written in bold red font. Abbreviations: CM—chorismate mutase; PPA-AT—prephenate aminotransferase; GOGAT—glutamine oxoglutarate aminotransferase; ADH—arogenate dehydrogenase; ADT—arogenate dehydratase; PAL—phenylalanine ammonia-lyase; C4H—cinnamate-4-hydroxylase; C3H—4-coumarate 3-hydroxylase; 4CL—4-coumarate-CoA ligase; HCT—hydroxycinnamoyltransferase; TYDC—tyrosine decarboxylase; NBS—norbelladine synthase; NR—noroxomaritidine reductase; N4OMT—norbelladine 4′-*O*-methyltransferase; CYP96T1—cytochrome P450 96T1 monooxygenase.

Recently, in attempts to respond to the demand of Amaryllidaceae alkaloids, substantial efforts have been made to improve the yields and biosynthesis efficiency of target molecules, including the application of metabolic engineering technologies. In contrast to the fast-growing knowledge about Amaryllidaceae alkaloids chemistry and pharmacological activities, very little is known about their biosynthesis, the responsible functional and regulatory genes, and the molecular mechanisms underlying its regulation [1,58,59]. Although rapid advances in omics technologies over the past few years have led to significant discoveries in Amaryllidaceae alkaloid biosynthesis, there are still many unknown details about their biosynthetic pathway (Figure 2). The biosynthesis of Amaryllidaceae alkaloids is a complex process and is a result of coordinated action of enzymes from different biosynthetic pathways of primary, secondary and specialized cell metabolism (Figure 2). According to Desgagne’-Penix [59], the biosynthetic pathway of Amaryllidaceae alkaloids could be provisionally divided into five stages: (1) Biosynthesis of the two aromatic amino acid L-phenylalanine and L-tyrosine, which are the building blocks of Amaryllidaceae alkaloids; (2) Formation of 3,4-dihydroxybenzaldehyde from phenylalanine, which is the aldehyde moiety of Amaryllidaceae alkaloids; (3) Formation of 4′-*O*-methylnorbelladine from norbelladine (a condensation product of tyramine with 3,4-dihydroxybenzaldehyde); (4) Formation of unstable intermediate products by specific phenol coupling of 4′-*O*-methylnorbelladine followed by a reduction step; (5) Biosynthesis of the different types of Amaryllidaceae alkaloids (galanthamine, lycorine, homolycorine, galasine, haemanthamine, plicamine, secoplicamine, narciclasine, pretazettine, crinine, cripowelline, graciline, montanine, ismine, cherylline and norbelladine types, as well as galanthindole, maritinamine and elwesine). The last stage involves very specialized enzymes which could be found in different plant species producing specific Amaryllidaceae alkaloids (Figure 2) [59,60].

The first stage involves the genes of the shikimate pathway, which are considered as genes from primary cell metabolism. Here, through a series of reactions, the chorismate produced via the shikimate pathway is converted into L-phenylalanine or L-tyrosine. The reactions are catalyzed by allosterically regulated enzymes: chorismate mutase (CM), prephenate aminotransferase (PPA-AT) or glutamine oxoglutarate aminotransferase (GOGAT), arogenate dehydratase (ADT) or arogenate dehydrogenase (ADH). The biosynthetic pathway is localized in plastids, and is part of the primary cellular metabolism, with the genes encoding the enzymes being very conservative [61].

The second stage involves the enzymes from the phenylpropanoid pathway (Figure 2). Here, the key enzyme is phenylalanine ammonia-lyase (PAL). PAL is a very important enzyme, since it redirects the carbon flux in the plant cell from primary to secondary metabolism, in this particular case to Amaryllidaceae alkaloid biosynthesis [59,62,63]. Several genes encoding PAL have been identified and characterized from different Amaryllidaceae species [58,59,60,64,65]. Recent phylogenetic analysis of identified Amaryllidaceae PALs showed that they can be divided into two main clusters named “PAL1” and “PAL2”, which share between 80 and 83% amino acid identities [59]. The transcript expression analyses of the genes from the two clusters suggest a different functional role for PAL1 versus PAL2. It has been demonstrated that PAL1 is expressed at similar levels in all plant parts (root, bulb, stem, leaf, flower), whereas PAL2 is expressed in the bulb [59,65,66]. Another important gene from the phenylpropanoid pathway, cinnamate-4-hydroxylase (C4H), has been recently cloned and characterized to be present as a one single transcript in Amaryllidaceae alkaloid producing plants [59,60,64,65,67]. The transcript expression analyses of C4H showed that the expression was highest in the bulbs of dormant and early germination stages, and it was also high in the stems, roots and bulbs in flowering plants [59,64,65,66].

The third stage involves a series of reactions, catalyzed by enzymes belonging to the tyramine pathway (tyrosine decarboxylase (TYDC)), and some specialized enzymes from Amaryllidaceae alkaloid biosynthesis (Figure 2). TYDC is an important enzyme, which catalyzes the production of tyramine from L-tyrosine [68]. It has been found that two transcript variants of TYDC are present in Amaryllidaceae alkaloid producing plants [59,65,66,69,70]. The phylogenetic analysis showed that Amaryllidaceae TYDCs can be divided into two main clusters: “TYDC1” and “TYDC2” [59]. The expression analyses suggested that TYDC1 may be involved in the synthesis of Amaryllidaceae alkaloids, whereas TYDC2 may be responsible for the production of other primary metabolites [59]. Recently, TYDC1 from *Lycoris aurea* (L’Hér) Herb. was cloned in *Escherichia coli* BL21 (DE3) and the expressed enzyme was characterized [69]. The specialized enzymes involved in stage three of Amaryllidaceae alkaloid biosynthesis are norbelladine synthase (NBS), noroxomaritidine reductase (NR) and norbelladine 4′-*O*-methyltransferase (N4OMT) (Figure 2). The NBS gene from *Narcissus pseudonarcissus* ‘King Alfred’ was recently cloned in *Escherichia coli* DH10β and the activity to produce norbelladine from tyramine and 3,4-DHBA of the expressed enzyme was demonstrated [67]. Recently, it was demonstrated that the enzyme NR from *Narcissus pseudonarcissus*, which catalyzes the formation of oxomaritinamine from noroxomaritidine, can catalyze the production of norbelladine from norcraugsodine, but with a 400-fold lower specific activity [58]. The gene encoding NR was cloned in *Escherichia coli* Rosetta II (DE3) and the expressed protein was characterized [71]. N4OMT has been identified and characterized in many Amaryllidaceae alkaloid producing plants [59]. The expression analyses showed that N4OMT is highly expressed in bulbs [59,65,70,72]. Interestingly, the *O*-methyltransferase found in *Lycoris aurea* (LauOMT1) was expressed at highest levels in flowers stalks and ovaries, and was demonstrated to have both *para*’ and *meta*’ *O*-methylation activities with a strong preference for the *meta*’ position methylation [73].

The fourth stage includes the specific reactions of phenol coupling of 4′-*O*-methylnorbelladine (*para-ortho’, ortho-para’* and *para-para’*), leading to formation of the main precursors of the different types Amaryllidaceae alkaloids [59,60]. Here, cytochrome P450 96T1 monooxygenase (CYP96T1) is the only known enzyme. CYP96T1 was the first enzyme with phenolcoupling activity, characterized in monocots [59]. CYP96T1 catalyzes the *para-para’* phenol coupling of 4′-O-methylnorbelladine, leading to the formation of (10bR, 4aS)-noroxomaritidine and (10bS, 4aR)-noroxomaritidine. The enzyme was also found to catalyze *para-ortho’* phenol coupling of 4′-*O*-methylnorbelladine, leading to the formation of nornarwedine but at a very low rate [74]. The expression analyses of CYP96T1 showed that the gene is highly expressed in the bulbs [59,65].

The genes and enzymes involved in the fifth stage of Amaryllidaceae alkaloid biosynthesis pathway remain unknown and should be identified in the near feature.

Obviously, a better understanding of Amaryllidaceae alkaloid biosynthesis is crucial so that the advantages of metabolic engineering can be applied in constructing effective plant or microbial systems able to produce a desirable molecule. The development of such expression systems will have a great impact on the cost of Amaryllidaceae alkaloids, their availability and sustainable production. Currently, the galanthamine is the only Amaryllidaceae alkaloid commercially applied in medicine, and the efforts are focused on increasing the yields of this acetylcholinesterase inhibitor in the plants and in vitro systems that can produce it. To achieve that goal, the methods of molecular engineering can be applied. Because the enzymes involved to the first stage of Amaryllidaceae alkaloids biosynthesis belongs to the primary cell metabolism, they are encoded by strongly conservative genes, and as such, are not suitable targets for manipulation. The possible engineering could be effective if made in the second, third and fourth stages of the pathway. This may include overexpressing of C4H, NBS and N4OMT, which are limiting enzymes in phenylpropanoid and specialized Amaryllidaceae alkaloids biosynthesis pathway. Another effective strategy could include silencing of the genes encoding PAL1 and TYDC2. This will increase the proportions of PAL2 and TYDC1 which will redirect the metabolic flow from production of other secondary metabolites to biosynthesis of Amaryllidaceae alkaloids. The new CRISPR-Cas9 technique could be applied to editing the genes, encoding NR and CYP96T1 and to engineer the enzymes by improving their specific activities to produce norbelladine and nornarwedine, respectively. Thill now, there is no enough information about the structural and regulatory genes involved into fifth stage of Amaryllidaceae alkaloids biosynthesis, and thus, we cannot suggest the optimal strategy for engineering this part of the pathway. As of the time of writing this review, there is no report on engineered plant or microbial system expressing target Amaryllidaceae alkaloids. It was suggested that a combination of enzyme catalyzed steps and chemical synthesis, or a combination of chemical synthesis of norcraugsodine as precursors and its conversion into nornarwedine by engineered microbial system expressing NR, N4OMT, and CYP96T1 could be effective for low cost production of galanthamine [75,76,77,78,79]. However, these processes must be implemented.

### 3.4. Optimization of the Biosynthetic Process

As stated in the Introduction, the optimization of the production process of Amaryllidaceae alkaloids is well documented, especially in the case of galanthamine [24,27,29,30,31,32,33]. It is clear that the main stages of such optimization are: optimization of the nutrient medium for maximal yields of the target alkaloids; selecting the appropriate cultivation system and improving its design; optimization of the environmental conditions of cultivation and process control, management and modeling.

Recently published research showed that the most appropriate approach to optimization of the composition of nutrient media for maximum biosynthesis of plant secondary metabolites by in vitro systems is the statistical optimization of the main nutrient components. However, the latest results presented by Ptak and co-workers [80] are evidence that it is expedient to perform a single-factor optimization of the influence of various independent variables on the yield of target alkaloids and on the basis of the results received to create an experimental matrix for the full-factor statistical optimization. In this way, the most appropriate type of carbon source [80], growth regulators [56,81], as well as biosynthetic precursors [82] can be identified and these independent variables can subsequently be included in the experimental matrix of multifactorial optimization. In our opinion, elicitation is one of the most effective approaches to optimizing the biosynthesis of Amaryllidaceae alkaloids and it is a mandatory step in a future technology for their production [32]. Important steps for effective elicitation are the experimental determination of the type of elicitor, its concentration and time of addition to the cultivation system, and these experiments should be carried out after the cultivation system is selected, as well as the composition of the nutrient medium and environmental conditions of cultivation are optimized. Elicitation is also a powerful tool for directing biosynthesis to one or another group of Amaryllidaceae alkaloids depending on the specific aim of the production process being developed [32].

The accumulated data in scientific literature clearly show that differentiated shoot type cultures are most suitable for the biosynthesis of Amaryllidaceae alkaloids (Table 1) [22,23,36,38,39,40,41,42,43,44,45], which creates problems with the development and optimization of cultivation systems [57]. However, enough data have recently been published about successfully developed cultivation processes based on shoots and plantlets. Rotary drum bioreactors [83,84,85], bubble column and mist bioreactor systems [86,87,88,89,90] have been successfully applied for the cultivation of a wide range of differentiated plant in vitro systems. The highest volume cultivation system that has been reported is a 500-L stirred tank for cultivation of *Stevia rebaudiana* Bertoni, but without mechanical agitation [91]. However, for the production of Amaryllidaceae alkaloids it seems that temporary immersion cultivation systems and modified bubble column bioreactors are the most appropriate. Temporary immersion cultivation systems are able to provide lower levels of shear stress and significantly reduce the shoots hyperhydricity. These cultivation systems were used for production of galanthamine and lycorine [82], as well as for galanthamine and related Amaryllidaceae alkaloids [31]. However, due to difficulties with inoculation of bioreactors and high labor costs, these types of cultivation systems are not suitable for the development of an industrial production process [27]. Based on these limitations, an illuminated bubble-column bioreactor with internal sections was developed in our laboratory. This bioreactor was implemented for the cultivation of *L. aestivum* shoots aiming galanthamine production. Results received from this experimental work revealed that more than 40% of alkaloids were secreted into the culture liquid [24] and this required further improvement of the design of the cultivation system to a two-phase bioreactor cultivation system. An essential part of this cultivation system was an external circulation column filled with adsorbent resin [33]. Using this system for the cultivation of *L. aestivum* shoots, metabolism of Amaryllidaceae alkaloids was redirected preliminary to galanthamine synthesis. The results obtained demonstrated that the two-phase bioreactor cultivation system is an important tool for improving the yields of secondary metabolites in plant in vitro system-based bioprocess and could be the basis for the development of large-scale Amaryllidaceae alkaloid production processes [24,33].

## 4. Conclusions and Future Prospects

Many of the known Amaryllidaceae alkaloids show remarkable biological activities and some of them, galanthamine for example, are already used in medicine. However, the limited amounts found in plants, in combination with the wide diversity in the chemical structures of these alkaloids render their production, isolation and purification very expensive.

Plant in vitro systems are an alternative approach to Amaryllidaceae alkaloid bioproduction. Nowadays, alkaloid yields are still too low and not attractive for commercial production despite the huge progress in the development of an integrated approach for their biotechnological production. It seems this disadvantage could be solved using metabolic engineering for constructing effective plant or microbial systems able to produce a desirable molecule. Possible solutions could be: (1) a combination of chemical synthesis of norcraugsodine as precursor and its conversion into nornarwedine by engineered microbial system; or (2) developing hybrid biosynthetic pathway for Amaryllidaceae alkaloids synthesis by yeasts or in vitro plant systems; or (3) developing hybrid bioprocess systems.

## Figures and Tables

**Table 1:** Recent reports on plant in vitro systems synthesizing Amaryllidaceae alkaloids.

Species	Type of In Vitro Systems	Amaryllidaceae Alkaloids	Accumulated Concentrations	References
*Narcissus pseuddonarcissus* cv. Carlton	callus shoots bulbs	Galanthamine Galanthamine Galanthamine	0–7 µg/g FW 40–130 µg/g FW 10–215 µg/g FW	[22]
*Narcissus tazetta* var. Meskin	callus	Galanthamine Lycorine	0.5–1.9 µg/g DW 1.2–21.5 µg/g DW	[38]
	bulblet	Galanthamine Lycorine	15–80 µg/g DW 731–1900 µg/g DW	
	roots	Galanthamine Lycorine	5.2 µg/g DW 131 µg/g DW	[39]
*Narcissus confusus*	shoots	Galanthamine Tazettine Haemanthamine	13.07 mg/g DW 0.75 mg/g DW 3.16 mg/g DW	[40]
*Hymenocallis littoralis*	callus	Lycorine	0.1–2.6 µg/g extract	[41]
*Rhodophiala bifida*	roots	Montanine	1.19 mg/g	[42]
	bulbs	Montanine	2.21 mg/g	
	leaves	Montanine	2.10 mg/g	
*Pancratium maritimum*	shoots	Lycorine	2.90 mg/g DW	[23]
	shoots	Haemanthamine Lycorine	900 µg/g DW 800 µg/g DW	[43]
*Leucojum aestivum*	calli	Galanthamine Lycorine	10–13 µg/g DW 10–30µg/g DW	[36,44]
	shoots	Galanthamine Lycorine	15–454 µg/g DW 100 µg/g DW	[36,44,45]
*Crinum moorei*	bulbs	Crinamidine Crinine Powelline Undulatine	130–200 mg/100g DW 25–60 mg/100g DW 25–50 mg/100g DW 25–80 mg/100g DW	[46]
*Hippeastrum vittatum*	bulbs	Montanine Pancracine 11-Hydroxyvitattine Vitattine	Data not presented	[47]
*Hippeastrum goianum*	bulbs	Lycorine	0.075–0.125 µg/mL	[48]
